# A Mouse Strain Defective in Both T Cells and NK Cells Has Enhanced Sensitivity to Tumor Induction by Plasmid DNA Expressing Both Activated H-Ras and c-Myc

**DOI:** 10.1371/journal.pone.0108926

**Published:** 2014-10-10

**Authors:** Li Sheng-Fowler, Wei Tu, Haiqing Fu, Haruhiko Murata, Lynda Lanning, Gideon Foseh, Juliete Macauley, Donald Blair, Stephen H. Hughes, John M. Coffin, Andrew M. Lewis, Keith Peden

**Affiliations:** 1 Division of Viral Products, Office of Vaccines Research and Review, Center for Biologics Evaluation and Research, Food and Drug Administration, Bethesda, Maryland, United States of America; 2 National Institute of Allergy and Infectious Diseases, Rockville, Maryland, United States of America; 3 National Cancer Institute, Rockville, Maryland, United States of America; 4 Frederick Cancer Research Facility, National Cancer Institute, Frederick, Maryland, United States of America; 5 Tufts University, Boston, Massachusetts, United States of America; University of Michigan School of Medicine, United States of America

## Abstract

As part of safety studies to evaluate the risk of residual cellular DNA in vaccines manufactured in tumorigenic cells, we have been developing *in vivo* assays to detect and quantify the oncogenic activity of DNA. We generated a plasmid expressing both an activated human H-*ras* gene and murine c-*myc* gene and showed that 1 µg of this plasmid, pMSV-T24-H-*ras*/MSV-c-*myc*, was capable of inducing tumors in newborn NIH Swiss mice. However, to be able to detect the oncogenicity of dominant activated oncogenes in cellular DNA, a more sensitive system was needed. In this paper, we demonstrate that the newborn CD3 epsilon transgenic mouse, which is defective in both T-cell and NK-cell functions, can detect the oncogenic activity of 25 ng of the circular form of pMSV-T24-H-*ras*/MSV-c-*myc*. When this plasmid was inoculated as linear DNA, amounts of DNA as low as 800 pg were capable of inducing tumors. Animals were found that had multiple tumors, and these tumors were independent and likely clonal. These results demonstrate that the newborn CD3 epsilon mouse is highly sensitive for the detection of oncogenic activity of DNA. To determine whether it can detect the oncogenic activity of cellular DNA derived from four human tumor-cell lines (HeLa, A549, HT-1080, and CEM), DNA (100 µg) was inoculated into newborn CD3 epsilon mice both in the presence of 1 µg of linear pMSV-T24-H-*ras*/MSV-c-*myc* as positive control and in its absence. While tumors were induced in 100% of mice with the positive-control plasmid, no tumors were induced in mice receiving any of the tumor DNAs alone. These results demonstrate that detection of oncogenes in cellular DNA derived from four human tumor-derived cell lines in this mouse system was not possible; the results also show the importance of including a positive-control plasmid to detect inhibitory effects of the cellular DNA.

## Introduction

The impetus for us to develop sensitive and quantitative animal models to assess the oncogenic activity of DNA arose because of the concerns that viral vaccines manufactured in certain types of neoplastic cells, such as those that were tumorigenic or were derived from human tumors, would pose an oncogenic risk to recipients of those vaccines. One source of this oncogenic risk would be the unavoidable presence of small quantities of residual cellular DNA in the vaccine and the likelihood that the genome of the neoplastic cell substrate would contain dominant activated oncogenes. While there has been no consensus as to whether residual cellular DNA from tumorigenic cells could transfer oncogenic activity to vaccine recipients [1], [2], few data were available concerning the activity of oncogenic DNA *in vivo*. Because our approach to evaluate risk involves extrapolation of data derived from sensitive and quantitative experimental systems [3], [4], we undertook a program to establish model systems to quantify the oncogenic activity of DNA *in vivo*. From such data, it was hoped that estimates of risk could be made.

Because the major source of the oncogenic activity in neoplastic cells would be activated cellular oncogenes, we have used cellular oncogenes rather than viral oncogenes for these studies. In our initial study [5], we generated expression plasmids for the T24 (activated) version of the human H-*ras* gene and the mouse c-*myc* gene, as these genes were known to transform primary rodent cells *in vitro* [6], [7] into cells that could form tumors in immunocompromised mice [6], [8]–[10]. The chosen promoter for these genes was the murine sarcoma virus (MSV) 5′ long terminal repeat (5′ LTR), and termination signals were the bovine growth hormone (BGH) poly(A) site followed by the MSV 3′ LTR. Inoculation of these plasmids by the subcutaneous route into adult and newborn NIH Swiss and C57BL/6 mice established that 1) tumors could be induced by direct introduction of DNA, 2) both oncogenes were required to induce tumors, 3) newborns were more susceptible than adults, and 4) NIH Swiss mice were more susceptible than C57BL/6 mice. The majority of tumors appeared between 4 and 9 weeks after inoculation, and cell lines established from the tumors expressed both the H-Ras and c-Myc oncoproteins. Analysis of the integration patterns of the DNA from tumor-cell lines demonstrated that most, if not all, of the tumors induced by the oncogenes were clonal. However, tumors were induced only at the highest dose of DNA (12.5 µg of each plasmid) with lower doses being insufficient [5].

To increase the sensitivity of the assay, several modifications to the original system were investigated. Because both oncogenes were required in the same cell for tumor induction, it was reasoned that placing both oncogenes on the same molecule would result in increased efficiency of tumor induction; this expectation was confirmed, as 1 µg of the dual-expression plasmid pMSV-T24-H-*ras*/MSV-c-*myc* was found to be oncogenic in newborn NIH Swiss mice [11]. In addition, because uptake of DNA was likely a rate-limiting step, we investigated whether transfection facilitators, compounds that increase DNA uptake *in vitro*, would increase the efficiency of tumor induction. Surprisingly, no transfection facilitator had any effect on tumor induction by DNA [11]. Furthermore, because we had found differences in the susceptibility of mouse strains as part of our exploratory studies, we evaluated various mouse strains, both immune competent and immune defective. In this paper, we report that the CD3 epsilon transgenic mouse, which is deficient in both T-cell and NK-cell functions [12], is the most sensitive mouse strain identified to date for the detection of oncogenic activity of DNA; amounts of DNA as low as 25 ng of the plasmid pMSV-T24-H-*ras*/MSV-c-*myc* were oncogenic in newborn CD3 epsilon mice. Importantly, when pMSV-T24-H-*ras*/MSV-c-*myc* was converted to linear molecules, this plasmid was found to be about thirty-fold more active, with 800 pg now inducing tumors in newborn CD3 epsilon mice. The availability of a sensitive *in vivo* system should make feasible the analysis of cellular and viral oncogenes following direct inoculation of DNA without the usual approach of expressing these oncogenes in cells *in vitro* followed by analyzing the phenotypes of these transformed cells *in vivo*. Such an approach might reveal hitherto unrecognized activities and functions of these oncogenes, which might also reveal novel mechanisms of oncogenesis. In addition, the identification of a highly susceptible mouse strain for the detection of the oncogenic activity of DNA prompted us to test the oncogenicity of DNA from four human tumors: HeLa, A549, HT-1080, and CEM. While tumors were induced in all animals inoculated with cellular DNA when the control pMSV-T24-H-*ras*/MSV-c-*myc* plasmid was co-injected, demonstrating that none of the cellular DNAs had inhibitory activity, no tumors were induced in mice that were injected with the tumor-cell DNA alone, which suggests that detecting activated oncogenes in cellular DNA might be problematic even with sensitive animal models such as the newborn CD3 epsilon mouse.

## Materials and Methods

### Oncogene expression plasmid

**Figure 1:**
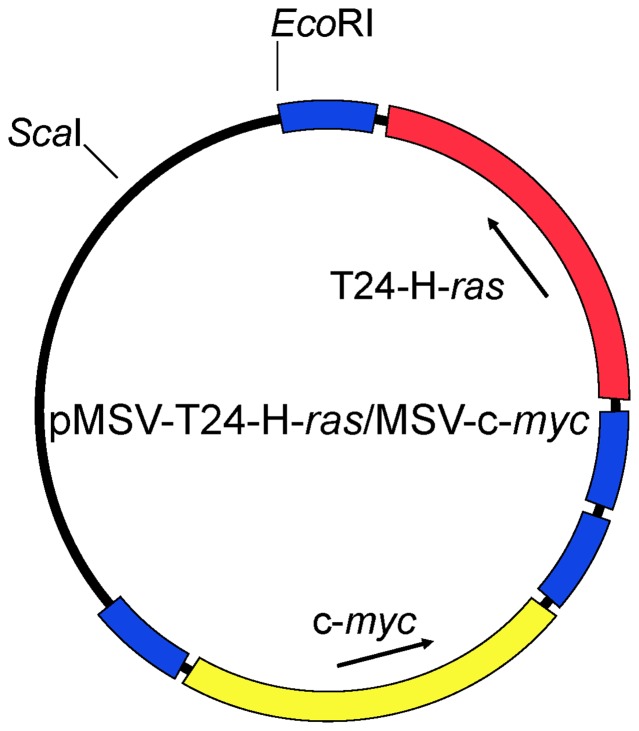
Structure of pMSV-T24-H-ras/MSV-c-myc. The human T24-H-*ras* gene is shown in red, the murine c-*myc* gene is shown in yellow, the MSV LTR elements are shown in blue.

The dual-expression plasmid pMSV-T24-H-*ras*/MSV-c-*myc* has been described [11]. Both oncogenes are expressed from their own promoters and terminators – the murine sarcoma virus (MSV) long terminal repeat (LTR) and the bovine growth hormone poly(A) site, respectively (Fig. 1).

### Animals and procedures

The CD3 epsilon transgenic mouse [B6;CBA-TgN(CD3E)26Cpt] [12] was obtained as a homozygous breeder pair from the Jackson Laboratories, Bar Harbor, ME, in 2002, and a breeding colony was established at the Center for Biologics Research and Evaluation (CBER). Mice were maintained under barrier cage isolation and with the antimicrobial drugs trimethoprim and sulphamethoxole added to the drinking water to 90 µg/mL and 450 µg/mL, respectively. Animals were housed in cages with food and water *ad libitum* and a 12-hour light/dark cycle. Protocols were approved by the CBER Institutional Animal Care and Use Committee.

Procedures for animal inoculations have been described [5], [11]. Briefly, various amounts of the dual-expression plasmid pMSV-T24-H-*ras*/MSV-c-*myc* DNA in PBS (total volume 50 µL) were inoculated *via* the subcutaneous route above the scapulae in adult and newborn mice using a 26-gauge needle and a 0.5-mL syringe. Newborns were injected within 72 hours of birth. For the inoculation of cellular DNA, 100 µg of DNA was inoculated with and without 1 µg of linear pMSV-T24-H-*ras*/MSV-c-*myc* DNA in 50 µL of PBS. Mice were monitored daily for general health and the development of tumors. When tumors reached 20 mm in any dimension, mice were euthanized.

### Establishment of cell lines from mouse tumors

Cells lines were established from minced tumor tissue explants. The tumor was washed with PBS or DMEM-10 medium [DMEM with 10% fetal bovine serum (FBS) and 2 mM glutamine] in a 6-cm dish. The liquid was removed, and the tumor was chopped into small pieces with sterile scissors. DMEM-10 (5 mL) was added, and the tumor tissue was transferred to a T25 flask. Tumor cells grew out from the explants. This method of cell-line establishment was found to be superior to tissue dispersal using trypsin digestion, which we had used in earlier studies [5], [11]. When the cells were near confluence (2 to 5 days, depending on the particular cell line), the tissue fragments were removed, and the cells were expanded in T75 flasks. The cell lines were frozen and cryo-preserved as described [5].

### Cell lines

All adherent cell lines were carried in DMEM-10 medium. The CEM cell line is a suspension cell line and is grown in RPMI-1640 medium with 10% FBS and 2 mM glutamine (RPMI-10). All human tumor cell lines were purchased from the American Type Culture Collection (Manassas, VA). HeLa cells were from a cervical carcinoma [13]–[15], A549 cells were from a lung adenocarcinoma [16], [17], HT-1080 cells were from a fibrosarcoma [18], and CEM cells were from a leukemia [19].

### Extraction of DNA from cell lines

Cells from the mouse tumor-cell lines and from HeLa, A549, and HT-1080 cells were harvested from T150 tissue-culture flasks by trypsinization and washed in PBS. CEM cells were propagated in suspension and were harvested by centrifugation and washed in PBS. Between 2×10^7^ and 1×10^8^ cells (1 to 4 flasks depending on the cell line) were used to extract genomic DNA by the Qiagen Blood and Cell Culture DNA kit using protocols supplied by the vendor (Qiagen Inc., Valencia, CA). DNA in TE (10 mM Tris-HCl, pH 7.5, 1 mM EDTA) was stored frozen or at 4°C.

### Southern DNA analysis

Genomic DNA (50 µg) from the tumor-cell lines was digested with restriction enzymes (*Bgl*II or *Bsr*GI) and analyzed for complete digestion by analytical agarose-gel electrophoresis. For the Southern analysis, 8 µg of each DNA were fractionated on a 1% agarose gel (10 cm×12 cm). After photographing, the gel was gently shaken at RT for 15 min in 0.25 N HCl to cause partial depurination of the DNA. The gel was then treated in 0.4 N NaOH for 30 min, followed by treating with 3 M NaCl, 0.4 M NaOH for 30 min, both with gentle shaking at RT, and the DNA was transferred for 4 h by downward capillary action to Amersham Hybond-N+ (GE Healthcare, Piscataway, NJ) in Transfer Buffer (3 M NaCl, 8 mM NaOH) using the Schleicher and Schuell TurboBlotter (Whatman Inc., Florham Park, NJ). The membrane was washed in 0.2 M sodium phosphate, pH 6.8, for 5 min. The DNA was cross-linked to the wet membrane using a UV Stratalinker 1800 (Stratagene, La Jolla, CA) with 120 mJoules/cm^2^ on Autocrosslink setting. The dried membranes were stored between two filter papers in plastic bags at 23–25°C.

Prehybridization and hybridization were done in glass roller bottles using a Hybaid Maxi 14 incubator. Membranes were prehybridized in 15 mL of PERFECTHYB PLUS (Sigma, St. Louis, MO) with 1.5 mg (0.1%) of denatured salmon DNA (Sigma; catalogue number D7656) at 68°C for 1–2 h. Probes were the gel-purified H-*ras* or c-*myc* genes (excised from the individual expression plasmids [5] using *Not*I plus *Bam*HI digestion) labeled using the North2South Biotin Random Prime Kit (Pierce, Rockford, IL). Denatured probes (450 ng; 30 ng/mL) were added to the prehybridization solution, and the membranes were hybridized overnight at 68°C in the glass cylinders in the HyBaid Maxi 14 incubator. Membranes were removed from the glass cylinders and washed once in 2× SSC (0.15 M NaCl, 0.015 M sodium citrate), pH 7.2, 0.1% SDS for 30 min at RT, twice in 0.5× SSC, pH 7.2, 0.1% SDS for 30 min each time at 68°C, and once in 0.1× SSC, pH 7.2, 0.1% SDS for 30 min at 68°C; all washes were with gentle shaking.

Probe detection was done according to the manufacturer's protocol (Pierce). Briefly, the hybridized membrane was incubated in blocking buffer with shaking for 15 min at 37°C and then in fresh blocking buffer with Stabilized Streptavidin-Horseradish Peroxidase Conjugate (1∶300) for another 15 min at 37°C with shaking. The membrane was washed 5 times at 37°C with 1 × wash solution (5 min each time) and incubated in substrate-equilibration buffer for 5 min with gentle shaking followed by addition of the chemiluminescent substrate working-solution buffer for 5 min without shaking. The membranes were exposed to X-ray film for times varying from a few seconds to several minutes. X-ray films were scanned using an Epsonperfection V500 photo scanner, and the images were saved as pdf files.

### Preparation of cell lysates and Western blot analysis

These were done using a modification of the methods described by Sheng *et al*. [5]. When the cells had reached 90–100% confluence, the medium was removed and the cells were lysed by addition 2% SDS, 1 mM EDTA, pH 8.0. The lysate was immediately heated at 95°C for 5 min to inactivate proteases and then stored at −80°C. The amount of protein was measured by the BCA method (BCA Protein Assay Kit; Pierce).

Prior to electrophoresis, samples were thawed, mixed with 5× SDS sample buffer (BioRad) to which dithiothreitol (DTT) was added to produce a final concentration of 100 mM, and heated at 95°C for 5 min. Cell lysates (2.5 µg of protein for H-Ras or 30 µg protein for c-Myc) were resolved by SDS-PAGE on Tris-glycine 4–20% gels (Invitrogen, Carlsbad, CA) and transferred to polyvinylidene difluoride (PVDF) membranes (Invitrogen) in 192 mM glycine, 25 mM Tris, pH 8.3, and 20% methanol. The PVDF membranes were blocked in 1x Tris-buffered saline (25 mM Tris-HCl, pH 7.4, 137 mM NaCl, 3 mM KCl) with 0.1% Tween (TBST) and 5% non-fat dry milk for 30 min at room temperature (RT), and then incubated overnight at 4°C with antibodies that were diluted in TBST with 5% non-fat dried milk. The rabbit polyclonal antibodies to H-Ras (C-20; sc-520) and actin (sc-1616) were purchased from Santa Cruz Biotechnology, Inc. (Santa Cruz, CA), and the c-Myc-specific antibody (9402) was from Cell Signaling Technology (Beverley, MA). Antibody dilutions of 1∶5,000 or 1∶2,000 were used for detection of H-Ras or c-Myc, respectively. The membranes were washed 3 times for 5 min each at RT with TBST and incubated for 1 h at RT with horseradish peroxidase (HRP)-conjugated anti-rabbit IgG (1∶5,000) followed by washing 3 times for 5 min each with TBST at RT. The proteins were detected by SuperSignal West Pico Chemiluminescent Substrate (Pierce). To confirm that equivalent amounts of protein were loaded in each well, the same membranes were washed in Restore Western Blot Stripping Buffer (Thermo Fisher) for 8 min at RT, washed three times for 5 min each time with TBST, blocked in TBST with 5% non-fat dried milk for 30 min at RT, and probed with the anti-actin antibody sc-1616 (Santa Cruz Biotechnology, Inc.) at a dilution of 1∶2,000; actin was detected as described above for the H-Ras and c-Myc proteins. The membranes were exposed to X-ray film for times varying from a few seconds to several minutes. X-ray films were scanned using an Epsonperfection V500 photo scanner, and the images were saved as pdf files.

### Immunofluorescence of cell lines

Cells (1×10^5^) were plated in four-well glass chamber slides (Nalge-Nunc; Rochester, NY) and incubated overnight in a CO_2_ incubator at 37°C. The next day, the medium was removed, and the cells were washed with PBST (PBS containing 0.1% Tween 20; Sigma) and fixed with freshly made 3.7% paraformaldehyde solution for 30 min at RT. [The 3.7% paraformaldehyde solution was made as follows. A 10% paraformaldehyde solution was made by mixing 1 g of paraformaldehyde with 10 mL water and 50 µL 2 N NaOH and boiling until the paraformaldehyde dissolved; this was diluted in PBS to make 3.7% paraformaldehyde in PBS.] After fixation, the cells were permeabilized by treating with ice-cold acetone/methanol (1∶1) on ice for 15 min. The slides were then washed with PBST three times for 5 min each with shaking at RT. The cells were incubated for 1 h at RT with 5% normal goat serum (Millipore, Billerica, MA) in PBST for blocking. After removal of the blocking buffer, the primary antibodies [anti-H-Ras mouse monoclonal antibody F235 (sc-29) and the anti-c-Myc rabbit polyclonal antibody C-19 (sc-788); Santa Cruz Biotechnology, Inc.] were added in 10% normal goat serum in PBST; both were used at 1∶100 dilution. The antibodies were incubated for 2 h at RT. The cells were then washed three times at RT for 5 min each time with PBST. The secondary antibodies were goat anti-mouse conjugated with Alexa Fluor 594 (for H-Ras) and goat anti-rabbit conjugated with Alexa Fluor 488 (for c-Myc), both from Invitrogen. Both secondary antibodies were used at 1∶2,500 dilution in PBST. The cells were incubated with the secondary antibodies for 1 h at RT and then washed three times for 5 min each time with PBST. The slides were mounted with VECTORSHIELD mounting medium (Vector Laboratories, Burlingame, CA), and the slides were visualized using an Olympus 1×51 microscope fitted for fluorescence. Pictures were taken using a 40x objective, and images were over-layered using Olympus CellSens software at a sensitivity of 800 ISO with a resolution of 680×512 for live images and 1360×1024 for snap images.

### Histopathology of tumors

At necropsy, tumors were excised and divided into equal parts for: 1) establishment of cell lines; 2) fixation in 10% neutral-buffered formalin for histopathology; and 3) frozen at −80°C for the subsequent isolation of DNA. Recently, an additional part of the tumor has been cryopreserved for immunohistochemistry. In addition, the following organs were fixed in 10% neutral-buffered formalin for histopathology: liver, kidney, spleen, heart, and lung. Routine sections of fixed tissues were trimmed, processed, embedded in paraffin blocks and sectioned at 5–7 microns. Tissue sections were then stained with hematoxylin and eosin and coverslipped in preparation for microscopic evaluation by the pathologist. Histology was performed by American Histo Labs, Inc. (Gaithersburg, MD). The tissue sections were examined by light microscopy. The images were taken using an Olympus microscope at 40x magnification.

## Results

### Characterization of the oncogenic activity of pMSV-T24-H-*ras*/MSV-c-*myc*

Our earlier work had shown that subcutaneous inoculation of plasmids expressing the human T24-H-*ras* oncogene and mouse c-*myc* proto-oncogene, each under control of the murine sarcoma virus (MSV) long terminal repeat (LTR), induced tumors in both adult and newborn immunocompetent mice, with newborns being more sensitive than adults, although the efficiency of tumor induction was low [5]. Because both oncogenes need to be present in a single cell to effect neoplastic transformation, the efficiency of tumor induction was increased twenty fold by incorporating both oncogene-expression cassettes on the same plasmid [11]. The structure of this dual-expression plasmid pMSV-T24-H-*ras*/MSV-c-*myc* is shown in Fig. 1. Using this plasmid, we assessed a number of both immune-competent and immune-compromised mice to identify a strain more sensitive to tumor induction by DNA than the NIH Swiss mouse. While several inbred strains (*e.g*., C3H, CBA, BALB/c) were found to have somewhat increased efficiency of tumor induction by the *ras/myc* plasmid compared with NIH Swiss mice (unpublished observations), the improvement in sensitivity was not sufficient to establish an assay for DNA oncogenicity.

### Studies with the CD3 epsilon mouse

**Figure 2:**
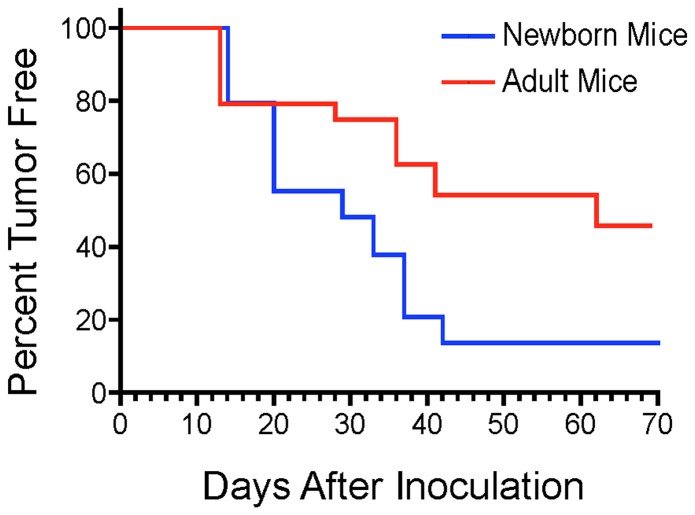
Kinetics of tumor appearance. Adult (4 weeks) and newborn (1 to 2-day old) mice were inoculated SC with 25 µg pMSV-T24-H-*ras*/MSV-c-*myc* DNA and followed for tumor appearance. Tumors were visible when they reached approximately 5 mm in diameter and were scored at that time. Observation was for a total of 6 months. Data were plotted according to the Kaplan-Meier estimator using GraphPad Prizm 5 software. The *p* value for the statistical equivalence of the two curves was 0.0063.

**Figure 3:**
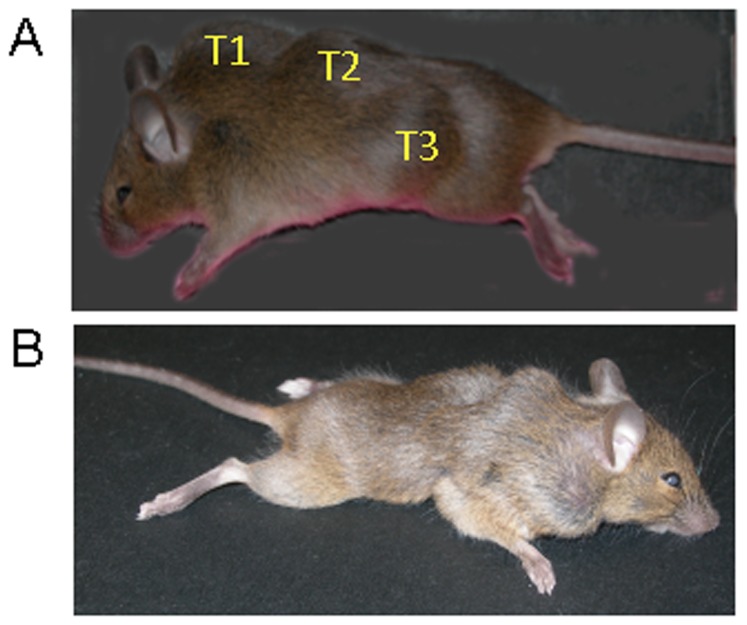
Multiple tumors induced in newborn CD3 epsilon mice. **A**. Three tumors evident in a newborn CD3 epsilon mouse that was inoculated SC with 25 µg of circular pMSV-T24-H-*ras*/MSV-c-*myc* DNA. **B**. Seven tumors evident in a CD3 epsilon mouse inoculated SC with 1 µg of linear pMSV-T24-H-*ras*/MSV-c-*myc* DNA; some of the tumors were not apparent until necropsy.

As part of another study to detect oncogenic viruses, we had established in CBER a breeding colony of the CD3 epsilon transgenic mouse. This mouse contains more than 30 copies of the human CD3 epsilon gene and is defective in both T-cell function and NK-cell function [12]. Because this mouse was shown to be at least as sensitive as the nude mouse in tumorigenicity assays [20], we evaluated it for its sensitivity to tumor induction by DNA in an oncogenicity assay. In our initial experiment, 25 µg of circular pMSV-T24-H-*ras*/MSV-c-*myc* was inoculated by the subcutaneous (SC) route into adult and newborn CD3 epsilon mice. Tumors were observed in both adults and newborns with the earliest tumors appearing after about two weeks and the last tumor at nine weeks. Newborns were more sensitive than adults, with tumors being induced in 86% (25/29) of newborns and 50% (12/24) of adults. When these results were plotted on a Kaplan-Meier survival curve (Fig. 2), it was apparent that both the kinetics of tumor appearance and the tumor incidence in newborns were significantly different from those in adults (*p* value 0.0063). Furthermore, not only was the overall tumor incidence higher than we had seen in other mouse strains, particularly in adult mice, but we observed for the first time mice with multiple tumors in both the newborns and the adults. An example of a mouse with three tumors (T1, T2, and T3) that was inoculated as a newborn is shown in Fig. 3A.

To determine the sensitivity of the CD3 epsilon mouse to tumor induction by oncogenic DNA, dose-response studies were done. Because newborn mice were more sensitive than adults (Fig. 2), only newborns were assessed. To address the variability we had observed in multiple assays in various studies, several dose-response experiments were carried out, and the results from each were pooled. As shown in the Table 1, tumors were induced with amounts of pMSV-T24-H*-ras*/MSV-c*-myc* DNA down to 25 ng.

**Table 1:** Dose-response data in CD3 epsilon mice inoculated with circular or linear pMSV-T24-H-*ras*/MSV-c-*myc*.

Amount of DNA (ng)	Circular DNA		Linear DNA	
	Number of Mice with Tumors	Percentage	Number of Mice with Tumors	Percentage
25,000	64/110	58.2	ND	ND
8,000	42/89	47.2	ND	ND
2,500	33/92	35.9	17/25	68.0
800	25/90	27.8	35/47	74.5
250	11/97	11.3	31/54	57.4
80	0/40	0	18/45	40.0
25	2/52	3.8	19/52	36.5
8	0/50	0	6/49	12.2
2.5	ND	ND	6/50	12.0
0.8	ND	ND	3/41	7.3
0.25	ND	ND	0/48	0

ND: Not done.

### Comparison between circular and linear plasmid

We next investigated whether lower amounts of oncogenic DNA could be detected by introducing the dual-expression plasmid as a linear molecule. Although studies had demonstrated that linear polyoma virus DNA was more oncogenic than its circular form [21], [22], and we had shown that linear retroviral DNA was more infectious than circular DNA *in vitro* [23], studies with intramuscular inoculation of DNA expression plasmids as DNA vaccines *in vivo* had demonstrated that circular DNA elicited stronger immune responses than did linear DNA [24], [25] and persisted for most of the lifetime of the mouse [26]. Therefore, it was not *a priori* predictable which form of DNA would be more oncogenic.

**Figure 4:**
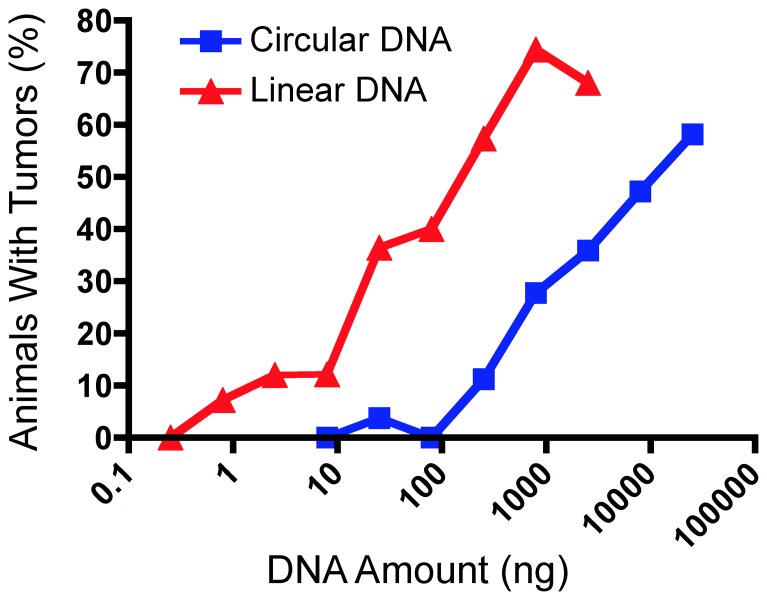
Linear DNA is more oncogenic than circular DNA. Data from the Table 1 were plotted using GraphPad Prizm 5.

To convert the circular plasmid into linear molecules, pMSV-T24-*H-ras*/MSV-*c-myc* was cleaved with either *Sca*I or *Eco*RI, enzymes that each has a single site in the plasmid. We tested both types of linear molecules, since the *Eco*RI site is adjacent to the 3′ LTR for the T24-H-*ras* expression cassette and cleavage at this position might affect its activity, while the *Sca*I site is located several hundred base pairs from the LTR. Because no differences were found in the oncogenic activity of either the *Eco*RI-digested or the *Sca*I-digested plasmid (data not shown), the data could be combined. As can be seen from the Table 1, linear DNA was more oncogenic than circular DNA, with amounts of DNA down to 800 pg inducing tumors. Furthermore, mice with multiple tumors were found more frequently when linear *ras*/*myc* DNA was inoculated compared with circular DNA. A mouse with seven tumors is shown in Fig. 3B. The results of the studies with circular and linear DNA are shown graphically in Fig. 4, where a clear dose-response relationship between the amounts of *ras*/*myc* DNA inoculated and tumor incidence is evident with both linear and circular molecules. Circular *ras/myc* plasmid DNA induced tumors at levels down to 25 ng DNA, while linear *ras/myc* DNA was active down to 800 pg.

In an analogous way to expressing the tumorigenicity of cells as the tumor-producing dose with a 50% endpoint or TPD_50_ [27]–[29], we have applied the data in the Table 1 and Fig. 4 to derive an analogous oncogenic dose 50% endpoint, or OD_50_. Analysis of the data using the Spearman-Karber method yields an OD_50_ of ∼5,000 ng for circular DNA in the newborn CD3 epsilon mouse, and an OD_50_ of ∼130 ng for linear DNA in this system. Thus, using either the endpoint estimate to compare the sensitivity of linear with circular DNA (800 pg *vs*. 25 ng) or the OD_50_ estimate (130 ng *vs*. 5 µg), linear DNA is about 30-fold more active than circular DNA in this system.

### Analysis of the DNA of cell lines established from tumors

To determine if the tumors were clonal and if the amount and site of integration of the *myc* and *ras* genes were different in tumors induced by circular or linear DNA, Southern hybridization analysis of DNA of cell lines established from tumors was carried out. Cell lines from 16 tumors induced by circular DNA and 13 lines from tumors induced by linear DNA were analyzed. DNA from each of the tumor-cell lines was digested with *Bgl*II, and the Southern membranes were probed with either the c-*myc* or H*-ras* genes. Since *Bgl*II cleaves the plasmid once, the integration pattern provides a straightforward way of determining whether the tumors were derived independently.

**Figure 5:**
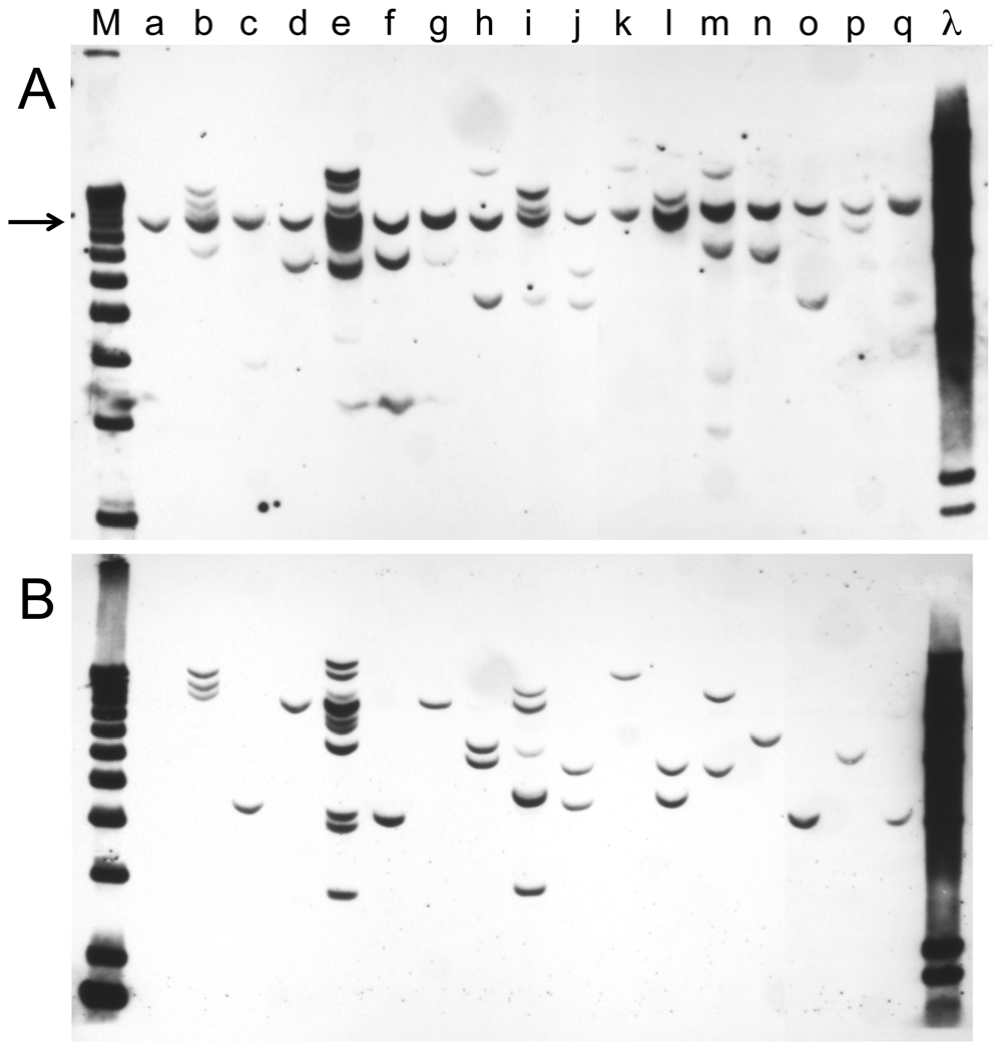
Integration patterns of the *myc* and *ras* genes in cell lines derived from tumors induced by circular pMSV-T24-H-*ras*/MSV-c-*myc*. DNA was isolated from tumor cell lines, digested with *Bgl*II, an enzyme that cuts the plasmid once, transferred to a membrane, and probed with a *myc* probe (**A**) or a *ras* probe (**B**). Lane a is mouse NIH/3T3 DNA; lanes b to q represent different tumor cell lines. Lanes e, f, and g are from three tumors found on a single mouse. Marker lanes are M (1 kb ladder) and λ (Lambda DNA digested with *Hind*III). The position of the endogenous mouse c-*myc* gene is indicated by the arrow.

Cell lines from tumors induced by circular DNA are shown in Fig. 5. In Fig. 5A, the membrane is probed with the c-*myc* gene, while in Fig. 5B, it is probed with the H-*ras* gene. Lanes (a) contain DNA from NIH/3T3 cells. As we reported previously, the endogenous c-*myc* gene is detected by the homologous mouse c-*myc* probe, whereas the human H-*ras* probe does not detect the mouse H-*ras* gene, at least at the hybridization stringency used [5]. No two cell lines have the identical pattern of hybridizing bands, indicating that all of the tumors were independently derived. In many cases, one or a few bands were present (lanes c, d, f, g, h, j, k, l, n, o, p, q), strongly suggesting that these tumors are clonal. In other cases, several bands were present (lanes b, e, i, and perhaps m), making a determination of tumor clonality impossible without additional studies, such as single-cell cloning of the tumor cell lines, as was done in an earlier study [5].

**Figure 6:**
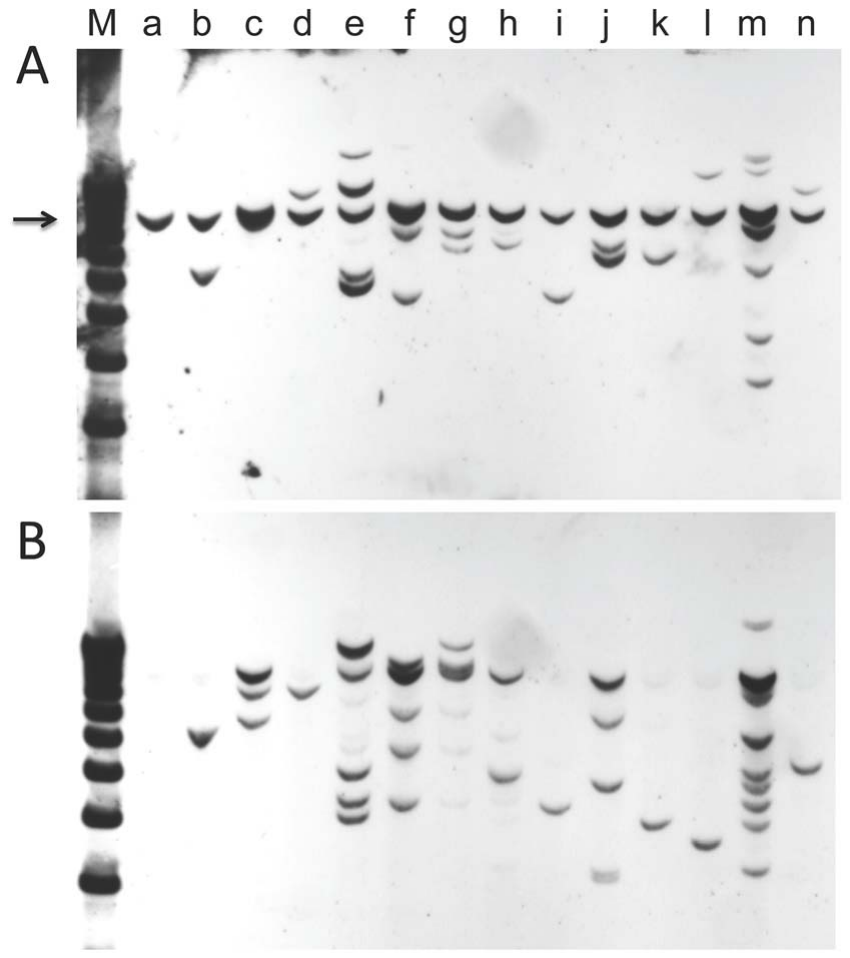
Integration patterns of the *myc* and *ras* genes in cell lines derived from tumors induced by linear pMSV-T24-H-*ras*/MSV-c-*myc*. DNA was isolated from tumor cell lines, digested with *Bgl*II, an enzyme that cuts the plasmid once, transferred to a membrane, and probed first with a *myc* probe (**A**), and then (after stripping the membrane) with a *ras* probe (**B**). Lane a is mouse NIH 3T3 DNA; lanes b to n represent different tumor cell lines. The position of the endogenous mouse c-*myc* gene is indicated by the arrow.

A similar conclusion can be made with the cell lines established from tumors inoculated with linear *ras*/*myc* plasmid (Fig. 6A and B), although the clonality of the tumors is not so obvious, since the amount of *ras/myc* DNA present in these cell lines is higher than in the cell lines established from tumors induced by the circular plasmid. Seven cell lines (lanes b, d, h, i, k, l, n) are likely derived from tumors that were clonal, while for others (lanes c, e, f, g, j, m), clonality cannot be assigned.

**Figure 7:**
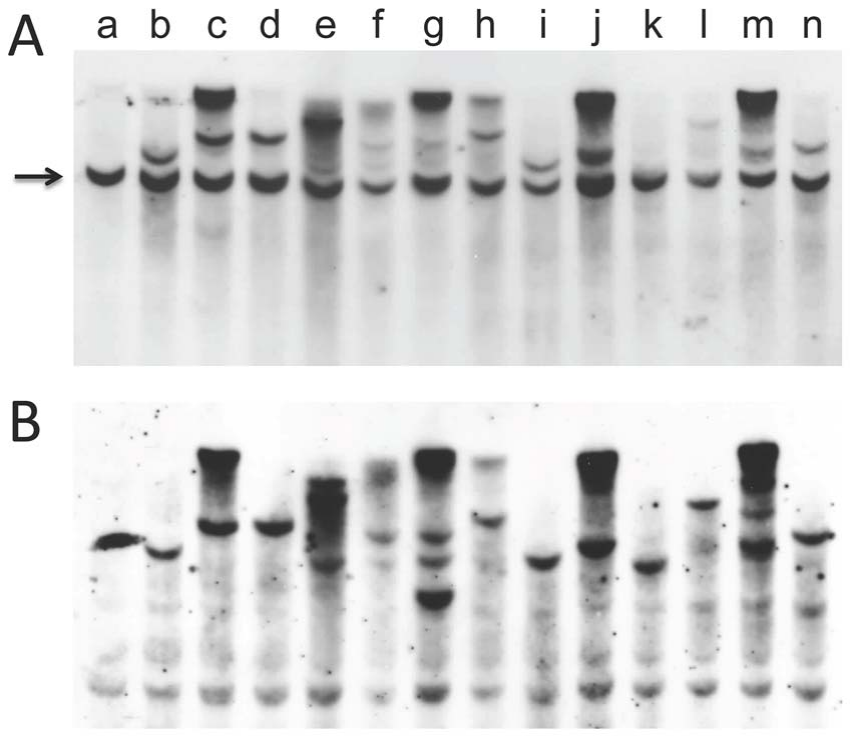
Analysis of the integration sites of the *myc* and *ras* genes in cell lines derived from tumors induced by linear pMSV-T24-H-*ras*/MSV-c-*myc*. DNA was isolated from tumor cell lines, digested with *BsrGI*, an enzyme that does not cut the plasmid, transferred to a membrane, and probed with a *myc* probe (**A**), or with a *ras* probe (**B**). Lane a is mouse NIH/3T3 DNA; lanes b to n represent different tumor cell lines. The position of the endogenous mouse c-*myc* gene is indicated by the arrow. [The apparent band in lane a in Fig. 7B is background.]

Because introduced DNA often become ligated and integrate at a single site, an alternative way to assess tumor clonality is to determine the integration pattern using restriction enzymes that do not cleave the *ras/myc* plasmid. A Southern hybridization analysis of tumor cell-line DNA following *Bsr* GI digestion and probing with c-*myc* is shown in Fig. 7A and probing with H-*ras* is in Fig. 7B. In this analysis, 6 cell lines (lanes b, d, i, k, l, and n) appear to have a single integration site, and two others (lanes f and h) may have a single integration site.

### Expression of H-Ras and c-Myc proteins

**Figure 8:**
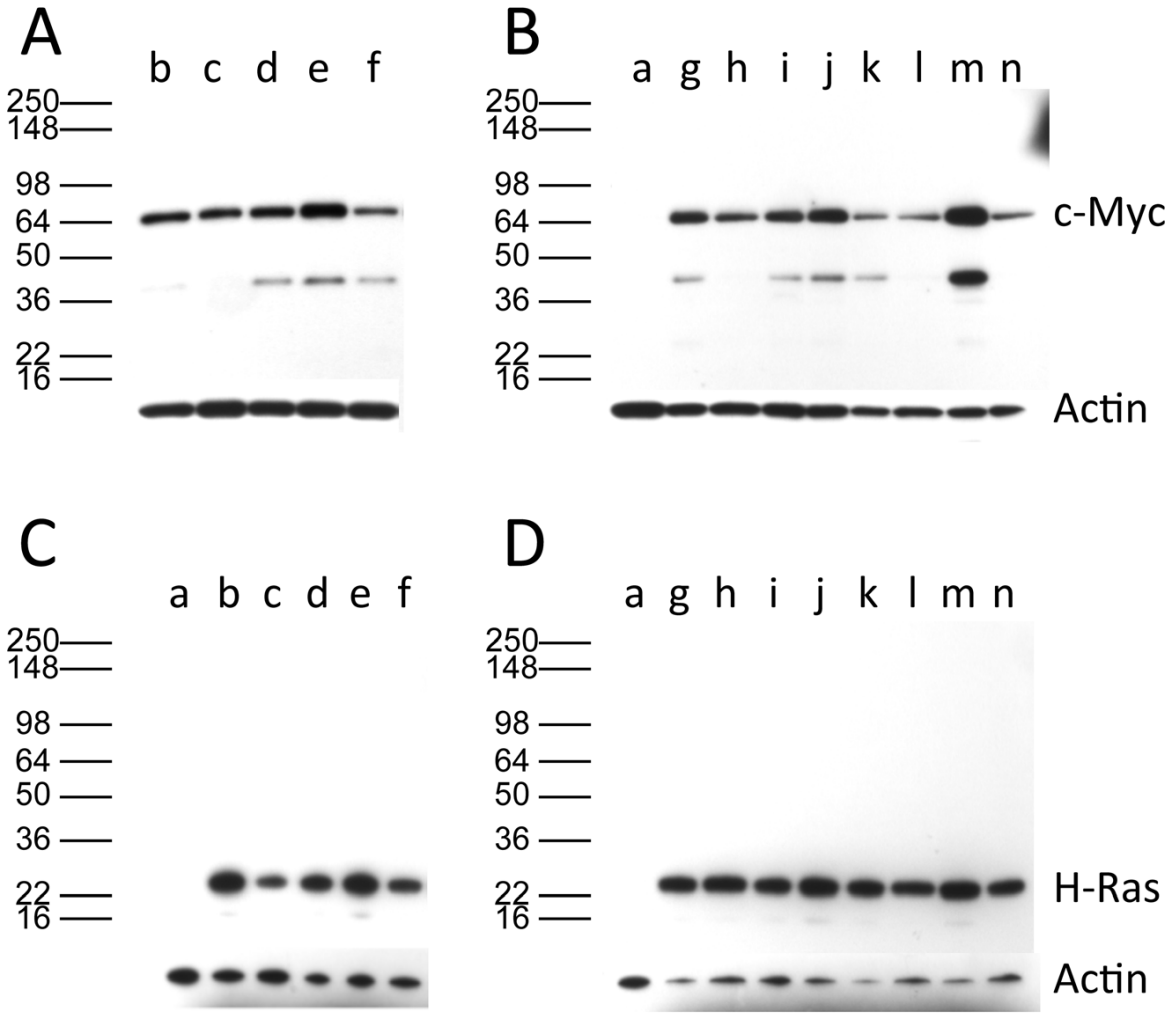
All tumor cell lines expressed both the H-Ras and the c-Myc proteins. Lysates of the tumor cell lines corresponding to 2.5 µg of protein for H-Ras detection and 30 µg of protein for c-Myc detection were resolved by SDS-PAGE on 4–20% gradient gels and the proteins revealed by western analysis. **A & B**: c-Myc. **C & D**: H-Ras. **A & C**: Lines from tumors induced by *Sca*I linear plasmid; **B & D**: Lines from tumors induced by *Eco*RI linear plasmid. Lanes a: NIH/3T3 mouse control cell line. Lanes b to n represent different tumor-cell lines; these lines correspond to the cell lines in Fig. 6 and Fig. 7. Membranes were subsequently reacted with an antibody to actin to assess protein loadings. The PVDF membranes were reacted with the anti-actin antibody sc-1616; a band migrating at approximately 43 kD is seen in all lanes.

To confirm that both the H-Ras and c-Myc oncoproteins were expressed in all tumor cell lines, two approaches were used – Western analysis and immunofluorescence. For the Western analysis, lysates were prepared from each cell line, and the proteins were fractionated by electrophoresis on 4 to 20% polyacrylamide gels. The separated proteins were transferred to PVDF membranes, and H-Ras and c-Myc were detected by antibodies specific to each protein followed by staining with second antibodies and chemiluminescence. As a control for protein loading, the membranes were stripped of the anti-H-Ras and anti-c-Myc antibodies and stained for cellular actin. Both the H-Ras and c-Myc oncoproteins were expressed in all of the tumor cell lines (Fig. 8), consistent with both genes being required for tumor induction. There were differences in the amount of oncoprotein expressed, but this did not correlate with the number of H-*ras* or c-*myc* genes in the tumor-cell lines.

**Figure 9:**
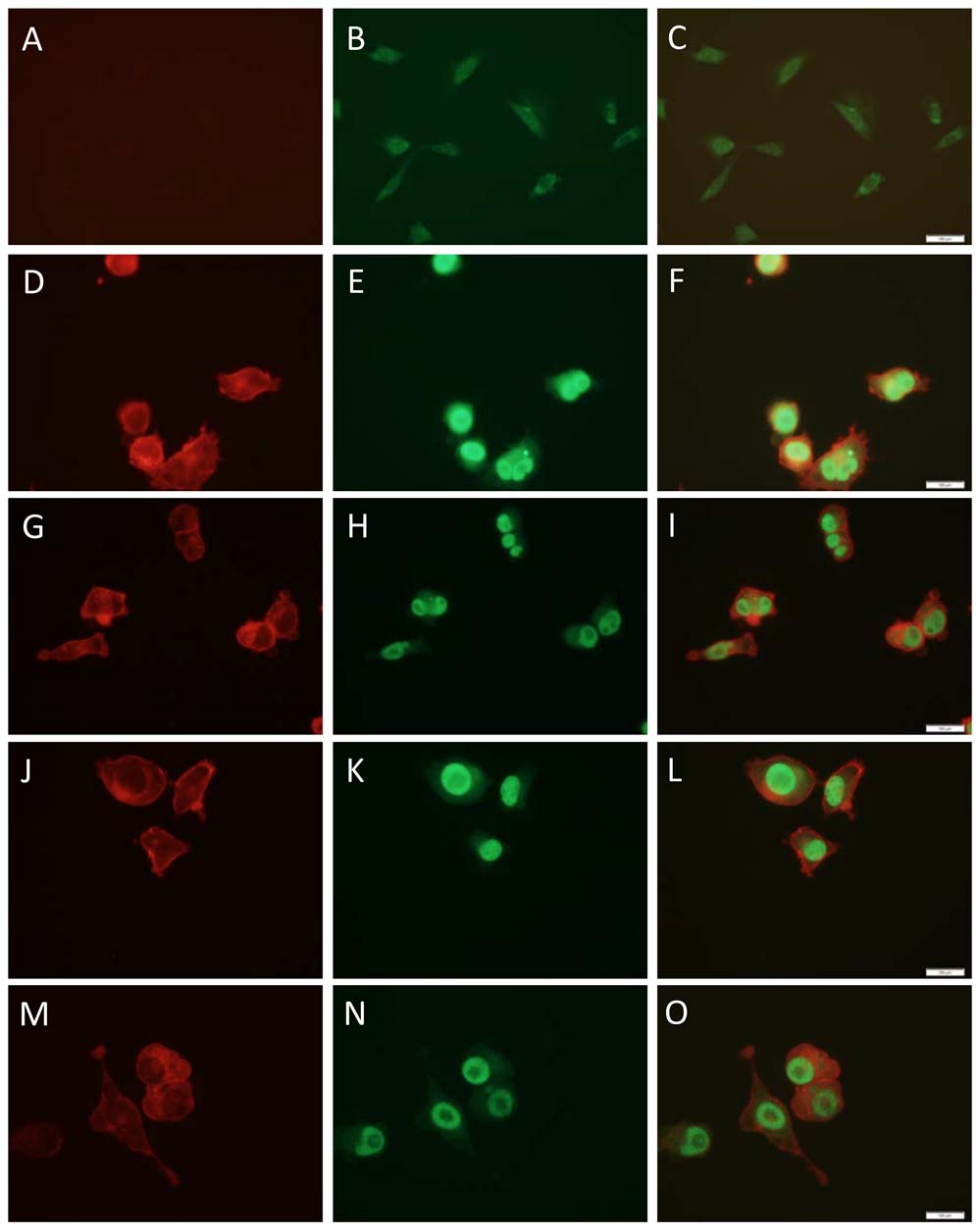
Localization of H-Ras and c-Myc in tumor-cell lines. Cells in chamber slides were fixed with 3.7% paraformaldehyde and permeabilized by treating with ice-cold acetone/methanol (1∶1) on ice for 15 min. The slides were then washed with PBS containing 0.1% Tween 20 (0.1%PBST) three times and blocked for 1 h with 5% normal goat serum. The anti-H-Ras mouse monoclonal antibody F235 (sc-29) and the anti-c-Myc rabbit polyclonal antibody C-19 (sc-788) were used at 1∶100 dilution. The antibodies were mixed and allowed to react overnight at 4°C. The second antibodies (at 1∶2500 dilution) were goat anti-mouse conjugated with Alexa Fluor 594 (for H-Ras) and goat anti-rabbit conjugated with Alexa Fluor 488 (for c-Myc). The slides were visualized using an Olympus 1×51 microscope fitted with fluorescence. Pictures were taken using a 40x objective, and images were over-layered using Olympus CellSens software at a sensitivity of 800 ISO with a resolution of 680×512 for live images and 1360×1024 for snap images. Panels **A**, **B**, **C**: NIH/3T3 cells. Panels **D**, **E**, **F** and **G**, **H**, **I** corresponded to two lines derived from tumors induced by circular *ras/myc* plasmid, while panels **J**, **K**, **L** and **M**, **N**, **O** corresponded to two lines derived from tumors induced by linear *ras/myc* plasmid. Panels **A**, **D**, **G**, **J**, **M**: anti-H-Ras antibody. Panels corresponded to two lines derived from tumors induced by circular *ras/myc* plasmid, **E**, **H**, **K**, **N**: anti c-Myc antibody. Panels **C**, **F**, **I**, **L**, **O**: merged images.

To determine the cellular location of the H-Ras and c-Myc proteins in the tumor-cell lines, cells plated onto coverslips were fixed in 3.7% paraformaldehyde, permeabilized with acetone/methanol, and reacted with antibodies to the oncoproteins. Since the species of these antibodies was different, second antibodies with specific fluorochromes could be used for double staining. Consistent with the known location of the oncoproteins, c-Myc (green) was in the nucleus, while H-Ras (red) was localized to the plasma membrane (Fig. 9). There was little staining with control NIH/3T3 cells for either c-Myc or H-Ras.

### Histopathology of the tumors

**Figure 10:**
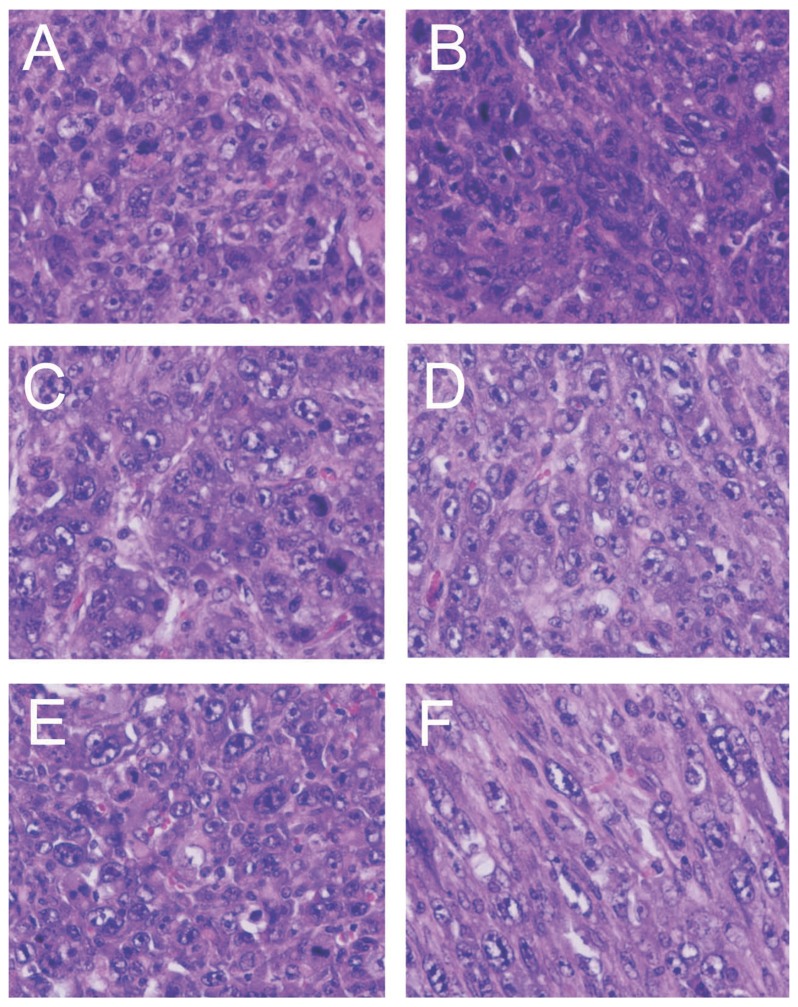
Histopathology of tumors induced by the *ras*/*myc* plasmid. Tumors induced by circular pMSV-T24-H-*ras*/MSV-c-*myc* (panels **A** and **B**), and tumors induced by linear pMSV-T24-H-*ras*/MSV-c-*myc* (panels **C**, **D**, **E**, and **F**). Tumors in panels **A** to **E** were undifferentiated sarcomas, while the tumor shown in panel F had the appearance of a differentiated fibrosarcoma.

In an earlier study, tumors induced by activated H-*ras* and c-*myc* in NIH Swiss and C57BL/6 mice were classified as undifferentiated sarcomas [5]. To determine whether this was also the case with tumors induced by activated H-*ras* and c-*myc* in the newborn CD3 epsilon mouse, we evaluated sections of 27 tumor tissues stained by hematoxylin and eosin. All but one tumor was an undifferentiated sarcoma; this exception was a differentiated fibrosarcoma. The neoplasms were composed of large pleomorphic cells with large nucleoli and the chromatin coarsely clumped and distributed primarily along the nuclear membrane (Fig. 10 A–E). The nuclear/cytoplasmic ratio was generally 1∶1 (normal ratio is 1∶4 to 1∶6), and the neoplasms have a high mitotic index of 3–4 per high power field. A few areas appeared to be differentiated with spindle-shaped cells with small nuclei (Fig. 10 F).

### Inoculation of cellular DNA derived from human tumors

**Figure 11:**
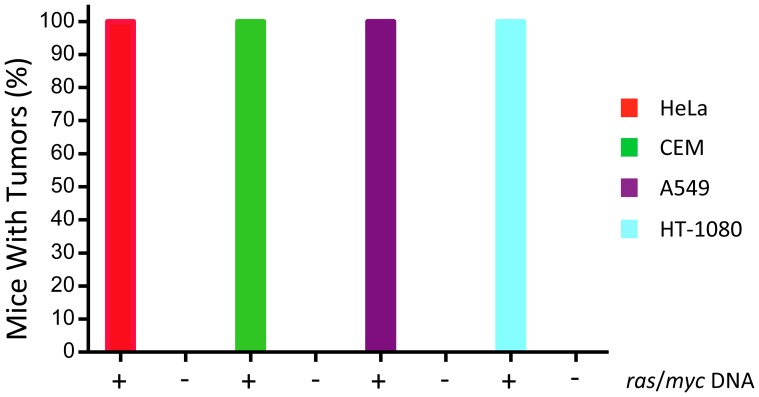
Evaluating the oncogenicity of DNA from cell lines established from four human tumors. Newborn CD3 epsilon mice were inoculated with 100 µg of cellular DNA from HeLa cells, CEM cells, A549 cells, or HT-1080 cells in the absence or presence of 1 µg of linear pMSV-T24-H-*ras*/MSV-c-*myc* DNA. Tumors were found in 14/14 mice inoculated with HeLa DNA plus *ras*/*myc* DNA but in 0/55 inoculated with HeLa DNA alone; tumors were found in 19/19 mice inoculated with CEM DNA plus *ras*/*myc* DNA but in 0/55 inoculated with CEM DNA alone; tumors were found in 13/13 mice inoculated with A549 DNA plus *ras*/*myc* DNA but in 0/53 inoculated with A549 DNA alone; and tumors were found in 5/5 mice inoculated with HT-1080 DNA plus *ras*/*myc* DNA but in 0/19 inoculated with HT-1080 DNA alone. Tumors appeared within 8 weeks, and all were at the site of inoculation.

To determine whether the newborn CD3 epsilon mouse is sensitive enough to detect activated oncogenes in cellular DNA, we isolated DNA from four human tumor-derived cell lines. These cell lines were established from four different types of human tumors. HeLa cells [13]–[15] were established from a cervical cancer initiated by human papillomavirus type 18 [30], A549 cells were from an adenocarcinoma of the lung [16], [17], HT-1080 cells were established from a fibrosarcoma [18], and CEM cells were established from a leukemia [31]. To control for the presence of possible inhibitors in the cellular DNA that might reduce or eliminate oncogenic activity, 100 µg of cellular DNA from HeLa cells, A549 cells, CEM cells, or HT-1080 cells were inoculated into newborn CD3 epsilon mice either in the presence of 1 µg of linear *ras*/*myc* plasmid DNA or in its absence. As can be seen from Fig. 11, none of the DNAs from the tumor-cell lines induced tumors in any of the mice during the six-month observation period, whereas tumors were induced in 100% of the mice that received tumor-cell DNA plus the positive-control *ras*/*myc* plasmid. These results confirmed that the cellular DNAs did not inhibit the oncogenicity of the positive control and demonstrated that these tumor-cell DNAs were not oncogenic even in this sensitive mouse.

## Discussion

The results described in this paper demonstrate that the CD3 epsilon mouse is sensitive to tumor induction by activated H-*ras* plus c-*myc* DNA. The results also confirm our earlier finding that the newborn mouse is more sensitive to this oncogenic insult than is the corresponding adult animal, which appears to be the case regardless of strain. Whether the sensitivity of the CD3 epsilon mouse is due to its defects in T-cell function and/or NK-cell function, or whether it is due to the particular genetic background, is unknown. With respect to the first question, we are using several other mouse strains with various immunological deficiencies to try to assess what components of the immune system might be contributing to the efficiency of tumor induction by the *ras*/*myc* DNA. With respect to the second issue, *viz*., the contribution of mouse strain background, this is more difficult to address, since the CD3 epsilon mouse is a hybrid between a CBA mouse and a C57BL/6 mouse and therefore there is no parental strain with which to investigate this issue. In earlier studies, we had evaluated adults and newborns of both the C57BL/6 mouse [5] and the CBA mouse (Sheng-Fowler, unpublished), and neither of these mouse strains was as sensitive to the *ras*/*myc* DNA-induced tumor formation as the CD3 epsilon mouse. Therefore, at present, the reason for the high sensitivity of the CD3 epsilon mouse is not known.

The reason that newborn mice are consistently more sensitive than adults regardless of the strain background is also unknown. One possible explanation is that newborn mice have increased numbers of proliferating cells compared with adults, and it is known that proliferating cells *in vitro* are more susceptible to oncogenic transformation by DNA than non-dividing cells [32]–[34]. In addition, the immune system is less well developed in newborn mice. The stage of development at which this higher sensitivity is lost is under investigation.

The finding that linear DNA was more oncogenic than circular DNA was not *a priori* predictable for several reasons. DNA vaccines are more efficacious *in vivo* as circular molecules [24], [25] and the DNA is retained longer after inoculation than is found with linear molecules [26]. While we found that linear cloned retroviral DNA was more infectious *in vitro* [23], we had also shown that circular SV40 DNA was more infectious *in vitro* than linear DNA [35]. Whether this reflects the difference between retroviral DNA and polyomavirus DNA is not known. However, circular mouse polyoma virus is more infectious in mice, and cleavage of the DNA eliminates infectivity [36]. Nevertheless, our finding confirms earlier studies on the oncogenicity of cloned mouse polyoma virus DNA, which showed that linear DNA was more effective than circular DNA in inducing tumors in newborn hamsters [21], [22], [36]. The reason that linear DNA is more oncogenic *in vivo* than circular DNA is unknown, but the more efficient integration of linear DNA compared with circular DNA is one possibility.

Our studies also demonstrated that most of the tumors are clonal, as was found in our previous studies [5], [11]. In addition, even when the *ras/myc* DNA induced multiple tumors in a single mouse, each tumor was independent and appeared to be clonal.

As found in our earlier studies, the tumors were predominantly undifferentiated sarcomas, which indicates that the initial cell transformed was mesenchymal in origin. This is consistent with the types of cells that are present in the subcutaneous tissue.

Because our original goal was to test whether cellular DNA could be oncogenic *in vivo*, we have made estimates as to what level of cellular DNA would be predicted to have equivalent oncogenic activity to our dual-expression plasmid in the newborn CD3 epsilon mouse. Due to the relative sizes of the pMSV-T24-H-*ras*/MSV-c-*myc* plasmid (approximately 8,000 bp) *versus* the haploid mammalian genome (approximately 3×10^9^ bp), the dose of cellular DNA that would be equivalent to 800 pg of pMSV-T24-H-*ras*/MSV-c-*myc* would be 800÷[(8×10^3^)÷(3×10^9^)] = 3×10^8^ pg, or 300 µg. This calculation does not take into account the number of potential dominant cellular oncogenes, which has been estimated to be 200 or more in the case of protein-coding oncogenes [37], although this may be higher if microRNA (miRNA) genes are considered. Because certain miRNAs can act as dominant oncogenes [38]–[41], the actual number of potential dominant oncogenes might be 300 or more. As we point out below, arithmetic extrapolations alone from our results with the *ras*/*myc* dual positive-control plasmid likely do not provide the complete picture.

Although only about 100 µg of cellular DNA can be tested in newborn mice, as 50 µL is the maximum volume that we routinely inoculate, it was nevertheless considered worth testing the oncogenicity of DNA from human tumor cell lines. Four cell lines from four human tumors of different types were chosen for the study: HeLa, A549, HT-1080, and CEM. HeLa cells were isolated in 1951 from an individual with cervical carcinoma [13] and contain between ten and fifty copies of HPV-18 DNA integrated at several sites in the human genome [42], [43]. A549 cells were isolated in 1972 from an individual with adenocarcinoma of the lung [16], [17] and were derived from alveolar basal epithelial cells. HT-1080 cells were isolated in 1972 from an individual with fibrosarcoma [18]; these cells are fibroblastic. CEM cells were isolated from a child with leukemia in 1965 [19]. All cell lines are tumorigenic in immunocompromised mice. One hundred micrograms of cellular DNA were inoculated into newborn CD3 epsilon mice with and without 1 µg of linear *ras*/*myc* positive-control plasmid. Addition of a positive control was considered critical so as to determine whether the presence of 100 µg of cellular DNA would either be inhibitory or obscure the activity of dominant oncogenes if they were present in the cellular DNA. The size of the inoculated DNA was between 10 and 20 kb. As we found (Fig. 11), far from inhibiting the positive control, 100 µg of cellular DNA from the human cells lines resulted in tumors being induced in 100% of the mice inoculated when 1 µg of linear *ras*/*myc* plasmid was included. This efficiency of tumor induction had not been observed in earlier experiments in newborn CD3 epsilon mice (*e.g*., Fig. 2 and Fig. 4). We are currently investigating the mechanism of this apparent stimulation of DNA oncogenicity, which is not limited to human tumor-cell line DNA and occurs with normal bovine and mouse DNA (unpublished results).

Nevertheless, the result was clear that none of the DNAs from these tumor cell lines induced tumors when inoculated in the absence of the positive-control *ras*/*myc* DNA. There are several possible reasons for this result. 1. The simplest explanation is that the amount of oncogenic DNA (the number of activated oncogenes) in these tumor cell lines is too low to be detected due to the limitation in the amount of DNA that can be inoculated in the newborn mouse. While the calculated amount of DNA that might be effective in inducing tumors is 300 µg, a value obtained above by extrapolating from the results with the positive-control plasmid, the actual concentration of functional dominant oncogenes in the tumor-cell DNA might be lower. 2. At least two dominant activated oncogenes are required to transform normal mouse cells into ones that can form a tumor in immunocompromised mice [6]. In our studies, two activated oncogenes, H-*ras* and c-*myc*, are required to induce a tumor in mice following direct inoculation [5]. Even if two or more dominant activated oncogenes are present in the tumor-cell DNA, it is unlikely that any two such oncogenes would be closely enough linked in the cell DNA such that both oncogenes would be taken up by the same cell. 3. The number of activated cellular oncogenes would likely be low even in tumorigenic cells or cells derived from tumors. For example, sequencing the genomes or exomes of human tumor-derived cell lines has revealed that only a few proto-oncogenes are activated by mutation even from highly malignant tumors [44]. From these types of studies it is known that the genome of A549 cells contains an activated K-*ras* gene [44] and the genome of HT-1080 cells contains an activated N-*ras* gene [45]–[47]. Interestingly, in assays to detect transforming activity of mammalian cellular DNA, such as the focus-forming activity in NIH/3T3 cells, DNA from A549 cells does not induce foci [48], while HT-1080 DNA does [45], [49]. 4. It is not clear that all classes of activated oncogene are able to induce tumors in the CD3 epsilon mouse. For example, even potent viral oncogenes, such as those from SV40 or high-risk human papillomavirus type 16, are only weakly active in the CD3 epsilon mouse or not active at all (unpublished results). 5. In those cases where tumor induction *in vivo* has been demonstrated following inoculation of DNA, the viral or cellular oncogenes are expressed from strong promoters [5], [22], [36], whereas the cellular oncogenes would be expressed from their own promoters and these would likely be subject to cellular control; some of them might also be silenced by DNA methylation and not be expressed. In any case, the level of expression from cellular promoters would likely be lower than that achieved from the MSV LTR used in our positive control. 6. A part of the mechanism that gave rise to the tumorigenic phenotype of the cell might be a consequence of inactivation of a tumor-suppressor gene. Unless this loss-of-function were due to the expression of a dominant-negative form of the tumor-suppressor protein, these types of mutations would not contribute to the oncogenic quotient of the DNA.

Whatever the reason, it is clear that even in this highly sensitive rodent system for the detection of the oncogenic activity of DNA, cellular DNA from lines derived from four human tumors failed to induce tumors. Current work is directed at determining whether cellular DNA could be capable of inducing tumors in experimental model systems. For this assessment, we are attempting to generate an optimal system. DNA has been isolated from several cell lines derived from tumors induced by the linear *ras*/*myc* DNA. Lines that have multiple copies of the *ras*/*myc* DNA integrated (see Fig. 7) will be selected. This type of DNA has the advantage that we know that the H-*ras* and c-*myc* oncogenes can induce tumors in the CD3 epsilon mouse and the two genes should be linked, thus providing the best opportunity to detect oncogenic activity of mammalian DNA.
